# Metchnikoff’s Ideas on Old Age and Disease

**Published:** 1905-12

**Authors:** 


### Abstracts and Selections.

### Metchnikoff's Ideas on Old Age and Disease.

An important exposition of this subject is made by L. Lematte (*Arch de Terap.,* December 15, 1904). Old age is a sort of disease allied to the changes that accompany atrophy. The same connective-tissue scleroses that occur in old people are also found in the various chemical intoxicants (e. g., alcohol and lead). The bacilli of tuberculosis and leprosy may produce a brittleness of the bones which is similar to that which is the result of senility. Old age is not the result of excessive use, but is an expression of a slow auto-infection. In the intestine is to be found the culture medium for those germs whose toxins are the source of senile atrophy. It is well known that birds have a longevity which is greater than that of the majority of mammalia. This is attributed to the fact that birds have no large intestine, in which hordes of bacteria may flourish. Even in old age birds preserve their youthful appearance and agility. The intestinal parasites secrete toxins which in some cases cause a destruction of hemoglobin and red-blood cells, sometimes poison the nervous centers, and sometimes cause an ulceration of the intestinal mucosa. It has been found that the lactic acid bacillus is a redoubtable foe of the putrefactive microbes. Moreover, a lactic acid ferment which is found in Bulgaria seems to be more active than those of other countries. Metchnikoff discovered this ferment in a preparation of milk consumed in large quantities by the Bulgarians, who are reputed for their longevity. It is suggested that this ferment be used in medicine. It precipitates casein, which it also partially digests, which fact explains the ease with which "Bulgarian milk" is digested. The lactic acid ferment found in this preparation of milk continues to live in the intestine, where it destroys unfriendly bacteria. This milk, containing the pure bacillus, has the consistency of white cheese. It has an agreeable, acidulous taste and contains neither alcohol nor carbonic acid. It is well tolerated by dyspeptics. It is indicated in fermentative dyspepsia. Inasmuch as Pawlow has shown that the organic acids are the best excitants of the intestinal digestive juices one may regard this form of milk as an excellent intestinal tonic. It may replace ordinary milk in the fetid diarrheas of typhoid fever and entritis. The peculiar ferment in the Bulgarian lactic acid bacillus splits up sugar into products which are harmless to the cells. It has been tried in the treatment of diabetes, with the result that the amount of glucose in the urine was diminished by two-thirds. Possibly this ferment may replace the distastes of yeast-cells and of raisins used in the treatment of microbic dermatoses. Bulgarian milk may be a useful adjuvant in the forced alimentation of cases of cancer and tuberculosis, used either alone, or mixed with yolk of egg, beef powder, etc.—*La Tribune Medicale, in Journal Med. and Science, Maine.*

